# Effects of Cannabinoid Exposure during Adolescence on the Conditioned Rewarding Effects of WIN 55212-2 and Cocaine in Mice: Influence of the Novelty-Seeking Trait

**DOI:** 10.1155/2016/6481862

**Published:** 2015-12-31

**Authors:** M. Rodríguez-Arias, C. Roger-Sánchez, I. Vilanova, N. Revert, C. Manzanedo, J. Miñarro, M. A. Aguilar

**Affiliations:** Unidad de Investigación Psicobiología de las Drogodependencias, Departamento de Psicobiologia, Facultad de Psicología, Universidad de Valencia, 46010 Valencia, Spain

## Abstract

Adolescent exposure to cannabinoids enhances the behavioural effects of cocaine, and high novelty-seeking trait predicts greater sensitivity to the conditioned place preference (CPP) induced by this drug. Our aim was to evaluate the influence of novelty-seeking on the effects of adolescent cannabinoid exposure. Adolescent male mice were classified as high or low novelty seekers (HNS and LNS) in the hole-board test. First, we evaluated the CPP induced by the cannabinoid agonist WIN 55212-2 (0.05 and 0.075 mg/kg, i.p.) in HNS and LNS mice. Then, HNS and LNS mice were pretreated i.p. with vehicle, WIN 55212-2 (0.1 mg/kg), or cannabinoid antagonist rimonabant (1 mg/kg) and were subsequently conditioned with WIN 55212-2 (0.05 mg/kg, i.p.) or cocaine (1 or 6 mg/kg, i.p.). Only HNS mice conditioned with the 0.075 mg/kg dose acquired CPP with WIN 55212-2. Adolescent exposure to this cannabinoid agonist increased the rewarding effects of 1 mg/kg of cocaine in both HNS and LNS mice, and in HNS mice it also increased the reinstating effect of a low dose of cocaine. Our results endorse a role for individual differences such as a higher propensity for sensation-seeking in the development of addiction.

## 1. Introduction

Drug addiction is a multifactorial disorder caused by the interaction of individual and environmental factors. Among the underlying factors that contribute to an enhanced predisposition to drug addiction are the existence of a vulnerable personality or phenotype [1–5], early exposure to drugs of abuse [6–8], and the presence of adverse environmental conditions such as exposure to stress [9–12]. In fact, evidence suggests that individual differences in susceptibility to addiction involve integrated neurocircuits underlying stress, reward, and behavioural inhibitory processes [9].

One of the most recognised factors facilitating the transition from voluntary, recreational drug use to dependence and addiction is exposure to drugs of abuse early on in life [13]. Adolescence is a critical developmental period characterized by immaturity of inhibitory control brain systems related with planning, evaluation of consequences, decision-making, and control of behaviour, such as the prefrontal cortex (PFC) [14, 15]. Moreover, the adolescent brain exhibits more plasticity and adolescents are more sensitive than adults to the rewarding effects of drugs and less sensitive to their aversive properties, all of which facilitate drug consumption at this age [14–17]. In fact, the characteristic behaviour of adolescents (impulsivity, emotional liability, increased risk-taking, enhanced novelty-seeking, etc.) that can favour drug use is due to this lack of prefrontal cortical maturation and hyperactivity of limbic structures involved in the processing of rewarding, emotional, and stressful stimuli, such as the nucleus accumbens (NAcc) and amygdala [14, 15].

Consumption of cannabis, the most used illegal drug, usually begins during adolescence, and an increase in the problematic use of this drug among adolescents has been reported in recent years [18–20]. Regular heavy use has more negative consequences at this early age than during adulthood, including enhanced vulnerability to develop dependence [21], suggesting that the adolescent brain is particularly vulnerable to the effects of cannabis exposure [22–24]. Furthermore, adolescent cannabis abuse seems to enhance vulnerability to later consumption of other drugs [25, 26]. Early onset of cannabis consumption has been shown to be a proximal trigger of later cocaine use [27–29] and increases the severity of cocaine withdrawal symptoms and relapse to cocaine dependence [30]. Similarly, in animal models, exposure to cannabinoid agonists (THC and CP 55940) during adolescence induces an upregulation of DAT in the caudate-putamen [31], increased self-administration of opioids, cocaine, and nicotine [32–37], and enhanced locomotor responses to cocaine [38]. In line with this, previous studies in our laboratory have demonstrated that preexposure to the cannabinoid agonist WIN 55212-2 increases the CPP induced by morphine [39] and the acquisition, persistence, and reinstatement of MDMA-induced CPP [40]. However, no previous studies have evaluated whether preexposure to cannabinoids during adolescence modifies the subsequent acquisition and reinstatement of the CPP induced by cannabinoids or cocaine.

Regarding the individual factors that contribute to drug addiction, differences in response to novelty and impulsivity that exist before the first experience of the drug have been related to differences in sensitivity to drug reward and vulnerability to addiction [1, 3, 4, 9]. In previous studies by our group we have observed that the novelty-seeking trait predicts greater sensitivity to the conditioned rewarding effects of cocaine [41, 42]. In particular, the hole-board test is a highly effective paradigm of novelty-seeking and predicts said sensitivity in adolescent male mice, since only high novelty seeker (HNS) adolescent mice have been shown to acquire the CPP induced by a low dose of cocaine, which is ineffective in inducing CPP in low novelty seeker (LNS) mice [42]. Moreover, we have observed a higher sensitivity of HNS to the conditioned rewarding effects of low doses of cocaine and MDMA in mice exposed to cocaine [43], ethanol [44], or MDMA [45]. However, the influence of the novelty-seeking phenotype on sensitivity to the rewarding effects of cannabinoids has not been studied to date.

Thus, the first aim of the present study was to evaluate the influence of the novelty-seeking phenotype on the sensitivity of adolescent mice to the rewarding effects of low doses of the CB1 agonist WIN 55212-2 in the CPP paradigm. We hypothesised that only HNS mice would acquire CPP after conditioning with a low dose of WIN 55212-2, as occurs with other drugs of abuse. The second aim was to study whether the stimulation or blockade of CB1 receptors during adolescence modifies the conditioned rewarding effects of WIN 55212-2 or cocaine and if such effects are modulated by the novelty-seeking phenotype. We expected preexposure to a cannabinoid agonist during adolescence to increase the CPP induced by low doses of WIN 55212-2 and cocaine and for this effect to be more pronounced in HNS mice.

## 2. Materials and Methods

### 2.1. Subjects

A total of 250 male mice of the OF1 strain were acquired commercially from Charles River (Barcelona, Spain) at 21 days of age. They were housed in groups of four in plastic cages (25 × 25 × 14.5 cm) for 5 days before experiments were initiated, under the following conditions: constant temperature (21°C), a reversed light schedule (white lights on 19.30–07.30 h), and food and water available ad libitum, except during behavioural tests. Animals were handled on each of the 3 days immediately prior to the preconditioning (Pre-C) phase in order to reduce their stress levels in response to experimental manipulations. Procedures involving mice and their care were conducted in conformity with national, regional, and local laws and regulations, which are in compliance with the European Directive 2010/63/EU.

### 2.2. Apparatus

The hole-board test was carried out in a square box (28 × 28 × 20.5 cm) with transparent Plexiglas walls and 16 equidistant holes of 3 cm in diameter in the floor. Photocells below the surface of the holes detected the number of times mice performed a head dip. Frequency of head dips was recorded automatically by the apparatus (CIBERTEC, SA, Spain).

For place conditioning, we employed twelve identical Plexiglas boxes with two equal sized compartments (length 30.7 cm, width 31.5 cm, and height 34.5 cm) separated by a grey central area (length 13.8 cm, width 31.5 cm, and height 34.5 cm). The compartments of these boxes had different coloured walls (black versus white) and distinct floor textures (fine grid in the black compartment and wide grid in the white one). Four infrared light beams in each compartment of the box and six in the central area allowed the position of the animal and its crossings from one compartment to the other to be recorded. The equipment was controlled using three PCs and MONPRE 2Z software (CIBERTEC, SA, Spain).

### 2.3. Drugs

Animals were injected i.p. with cocaine hydrochloride (Laboratorio Alcaliber SA, Madrid, Spain), WIN 55212-2 (Tocris, Biogen Científica, S.L., Madrid, Spain), or rimonabant (SR 141716A, Sanofi Recherche, Montpellier, France) in a volume of 0.01 mL/g. Control groups were injected with the physiological saline used to dissolve cocaine (NaCl 0.9%) or with Tween-80 (Sigma-Aldrich, Madrid, Spain), which was used to dissolve WIN 55212-2 and rimonabant (0.01%, 0.01 mL of Tween dissolved in 100 mL of saline). The doses of cocaine we administered were selected on the basis of previous studies showing that 1 mg/kg is a subthreshold dose for inducing CPP in naïve mice, while 6 mg/kg is effective in inducing CPP acquisition but not in producing reinstatement after extinction of CPP [41, 46]. Similarly, the doses of cannabinoid drugs administered were selected on the basis of previous studies on the effects of WIN 55212-2 in the CPP paradigm [39, 47] and on the effects of cannabinoid pretreatment on the CPP induced by other drugs of abuse [40]. Pretreatment of mice with 0.1 mg/kg of WIN 55212-2 is effective in increasing the CPP induced by MDMA [40], while 1 mg/kg of rimonabant specifically blocks CB1 receptors and does not act as an inverse agonist [48].

### 2.4. Procedure of Hole-Board Test

**Figure 1:**
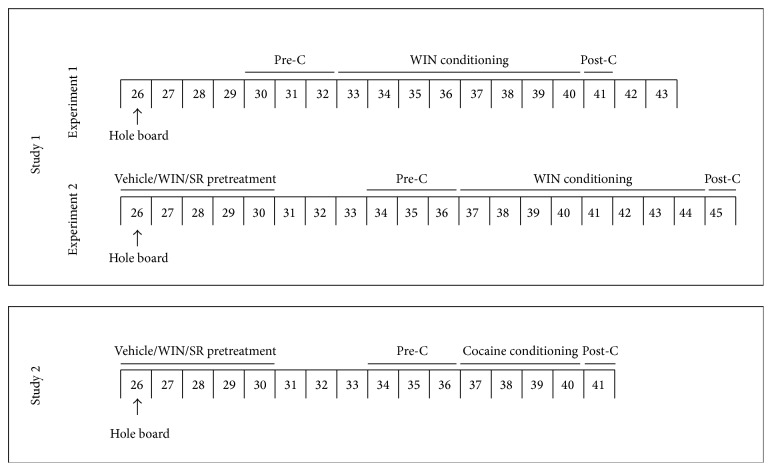
Timeline of experiments.

At the beginning of the test, mice were placed in the same corner of the box and were allowed to freely explore the apparatus for 10 min. The illumination in the experimental room consisted of four neon tubes fixed to the ceiling (light intensity of 110 lux at 50 cm above floor level). In all experiments, animals were first tested in the hole board on PND 26 (prior to any treatment) and defined as high novelty seekers (HNS) or low novelty seekers (LNS) according to whether the number of head dips they performed was higher or lower than the median of the group. We have previously used this median-split analysis to study the effects of novelty-seeking on the behavioural effects of different drugs of abuse [42–45]. In experiment 1, mice initiated the place conditioning procedure six days after the hole-board test (on PND 32). In the other experiments, given that mice received a pretreatment after being classified as HNS or LNS, the place conditioning procedure began two days after (on PND 34). Thus, the window of adolescence taken into account for the experiments included from PND 26 to PND 45 (see a timeline of the experiments in Figure 1).

### 2.5. CPP Procedure

Place conditioning, consisting of three phases, took place during the dark cycle. During the first phase, or preconditioning (Pre-C), mice were allowed access to both compartments of the apparatus for 15 min (900 s) per day for 3 days. On day 3, the time spent by the animal in each compartment during 900 s period was recorded. Animals showing strong unconditioned aversion for any compartment (less than 33% of the session time) were excluded from the rest of the procedure so that the CPP procedure was unbiased in terms of initial spontaneous preference [46, 47]. One compartment was paired with the drug and the other with the vehicle using a counterbalanced design such that half the animals in each group received the treatment in one compartment and the other half in the other compartment. After assigning compartments, no significant differences were observed between the time spent in the drug-paired and vehicle-paired compartments during the preconditioning phase. This is an important step in the experimental procedure that avoids any preference bias before conditioning.

In the second phase (conditioning), animals were conditioned with WIN 55212-2 or cocaine, as described in previous studies [46, 47]. In brief, in the case of WIN 55212-2, mice were treated with WIN 55212-2 immediately before confinement for 30 min to the drug-paired compartment (days 4, 6, 8, and 10) and with vehicle before confinement to the vehicle-paired compartment (days 5, 7, 9, and 11). In the case of cocaine, mice underwent two pairings per day on days 4, 5, 6, and 7, receiving an injection of physiological saline immediately before being confined for 30 min to the vehicle-paired compartment and receiving an injection of cocaine after an interval of 4 h, immediately before confinement to the drug-paired compartment.

During the third phase (postconditioning, Post-C), which took place on day 8 (in the case of cocaine) or day 12 (in the case of WIN 55212-2), the guillotine door separating the two compartments was removed and the time spent by the untreated mice in each compartment was recorded during a 900 s observation period. The difference in seconds between the times spent in the drug-paired compartment in the Post-C versus Pre-C test is a measure of the degree of conditioning induced by the drug. If this difference is positive, then the drug is considered to have induced a preference for the drug-paired compartment.

Groups showing CPP in Post-C underwent an extinction session every three days (on Mondays, Wednesday, and Friday) during which they were placed in the apparatus (without the guillotine doors separating the compartments) for 15 min until the time spent by each group in the drug-paired compartment was similar to that of Pre-C and differed from that of Post-C (Student's *t*-test). After extinction had been confirmed in an additional session, a reinstatement test was performed 15 min after administration of a priming dose (half of that used during conditioning) of the respective drug (0.0375 mg/kg of WIN 55212-2, 0.5 or 3 mg/kg of cocaine).

### 2.6. Experimental Design

In Study 1, two experiments were performed in order to study the influence of the novelty-seeking phenotype on the effects of cannabinoid exposure on the acquisition of the CPP induced by WIN 55212-2. In the first experiment, 60 male mice performed the hole-board test in order to be classified as HNS or LNS and were randomly assigned a drug treatment (0.075 or 0.05 mg/kg of WIN 55212-2). Thus, four groups of mice were formed according to novelty-seeking profile and the dose of WIN 55212-2 received during CPP conditioning (HNS+WIN 0.075, HNS+WIN 0.05, LNS+WIN 0.075, and LNS+WIN 0.05). The CPP procedure began on PND 32 and conditioning took place from PND 35 to PND 42. After the Post-C test, groups showing CPP underwent extinction and reinstatement tests. In the second experiment, 80 male mice performed the hole-board test in order to be classified as HNS or LNS and were randomly assigned a drug treatment (vehicle, 0.1 mg/kg of WIN 55212-2 or 1 mg/kg of rimonabant). Mice received one daily injection of their respective treatment for 5 days (PND 26–30) and, after an interval of 3 days without any treatment, underwent the CPP procedure following conditioning with the same dose of WIN 55212-2 (0.05 mg/kg). Thus, six groups of mice were formed according to novelty-seeking profile and the pretreatment received before conditioning with WIN 55212-2 (HNS-Veh-WIN, HNS-WIN-WIN, HNS+SR-WIN, LNS-Veh-WIN, LNS-WIN-WIN, and LNS-SR-WIN). The place conditioning procedure began on PND 34, and conditioning took place from PND 37 to PND 44.

Study 2 was performed in order to study the influence of the novelty-seeking phenotype on the effects of cannabinoid exposure on the acquisition of the CPP induced by cocaine. Eighty male mice performed the hole-board test and were defined as HNS or LNS and randomly assigned a drug treatment (vehicle, 0.1 mg/kg of WIN 55212-2 or 1 mg/kg of rimonabant). The animals received a daily injection of their respective treatment for 5 days (PND 26–30) and, after an interval of 3 days without any treatment, underwent the CPP procedure having been conditioned with the same dose of cocaine (1 mg/kg). Thus, six groups of mice were formed according to novelty-seeking profile and the pretreatment received before conditioning with cocaine (HNS-Veh-COC, HNS-WIN-COC, HNS+SR-COC, LNS-Veh-COC, LNS-WIN-COC, and LNS-SR-COC). Following the Post-C test, groups showing CPP underwent extinction and reinstatement tests. With the objective of corroborating the results obtained in the groups receiving pretreatment with WIN 55212-2 and conditioned with 1 mg/kg of cocaine, two additional groups were included in the procedure. Thirty mice performed the hole-board test in order to be classified as HNS or LNS and were then treated with 0.1 mg/kg of WIN 55212-2 for 5 days. After an interval of 3 days without any treatment, the mice underwent the CPP procedure having been conditioned with 6 mg/kg of cocaine (HNS-WIN-COC6 and LNS-WIN-COC6). In this study all the groups began the CPP procedure on PND 34, and conditioning took place from PND 37 to PND 40.

### 2.7. Statistical Analysis

Differences between the number of dips performed by HNS and LNS groups were analysed with Student's *t*-tests. To evaluate the influence of the novelty-seeking trait on the CPP induced by WIN 55212-2 (Study 1, experiment 1), data of the time spent by the animals in the drug-paired compartment were analysed by means of a mixed ANOVA with two between-subjects variables: “Novelty-Seeking,” with two levels (HNS and LNS), and “Treatment,” with two levels (WIN 0.05 and WIN 0.075), and one within-subjects variable: “Days,” with two levels (Pre-C and Post-C). To evaluate the effect of pretreatment with cannabinoid drugs on the subsequent CPP induced by WIN 55212-2 or cocaine in HNS and LNS mice (Study 1, experiment 2; and Study 2), data of the time spent in the drug-paired compartment were analysed with a mixed ANOVA with two between-subjects variables: “Novelty-Seeking,” with two levels (HNS and LNS), and “Pre-Treatment,” with three levels (Veh, WIN, and SR), and one within-subjects variable: “Days,” with two levels (Pre-C and Post-C). In the abovementioned experiments, extinction and reinstatement values in the groups showing CPP were analysed by means of Student's *t*-tests. To evaluate the effect of pretreatment of HNS and LNS mice with WIN 55212-2 on the subsequent CPP induced by 6 mg/kg of cocaine (additional groups of Study 2), data of the time spent in the drug-paired compartment were analysed with a mixed ANOVA with one between-subjects variable: “Novelty-Seeking,” with two levels (HNS and LNS), and one within-subjects variable: “Days,” with four levels (Pre-C, Post-C, Extinction, and Reinstatement). The time required for preference to be extinguished in each animal was analysed by means of the Kaplan-Meier test, with Breslow (generalized Wilcoxon) comparisons when appropriate. In all the ANOVAs, post hoc comparisons were performed with Bonferroni tests. Linear and logistic regression analysis was employed to determine the association between the level of novelty-seeking and the development of preference.

## 3. Results

**Figure 2:**
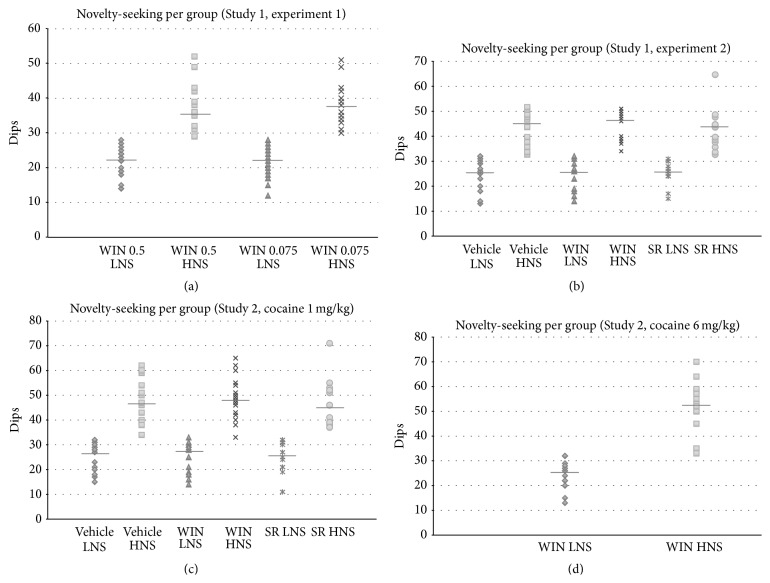
Novelty scores for each mouse, identified by group, in Study 1 ((a) experiment 1 and (b) experiment 2) and Study 2 ((c) mice conditioned with 1 mg/kg of cocaine and (d) mice conditioned with 6 mg/kg of cocaine).

The novelty scores for each mouse, identified by group, are represented in Figure 2. Although the distribution is not completely bimodal (some mice had a similar novelty-seeking score in LNS and HNS groups), they are clearly different with respect to the median scores. In all experiments, Student's *t*-tests showed significant differences between the number of dips performed by HNS and LNS groups (*p*s < 0.01).

### 3.1. Study 1

Study 1 explains the influence of the novelty-seeking phenotype on the effects of cannabinoid exposure on acquisition of the CPP induced by WIN 55212-2.

#### 3.1.1. Experiment 1

Experiment 1 is about the influence of the novelty-seeking phenotype on the sensitivity of mice to the rewarding effects of WIN 55212-2.

**Figure 3:**
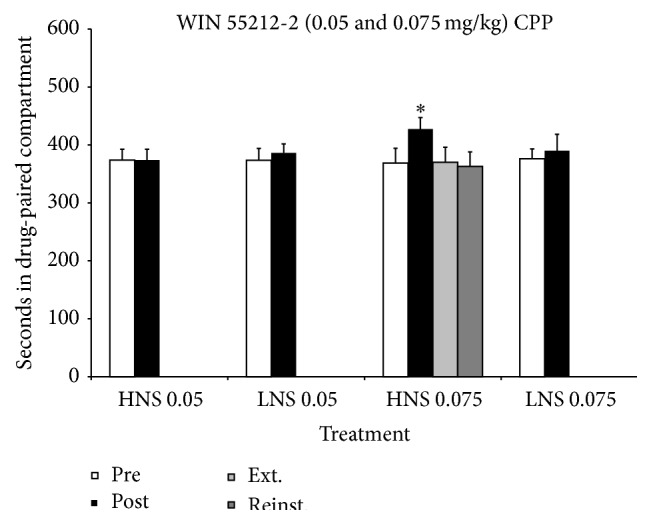
Effects of WIN 55212-2 (0.05 and 0.075 mg/kg) on the CPP paradigm in HNS and LNS adolescent mice (HNS 0.05, *n* = 12; LNS 0.05, *n* = 12; HNS 0.075, *n* = 13; LNS 0.075, *n* = 14). Bars represent time in seconds spent in the drug-paired compartment during preconditioning (white), postconditioning (black), the last extinction session (light grey), and reinstatement (dark grey). Values are mean ± SEM. ^*∗*^ *p* < 0.05, difference with respect to the preconditioning session.

The ANOVA of the data obtained with the mice conditioned with 0.05 and 0.075 mg/kg of WIN 55212-2 revealed that the interaction “Days × Treatment × Novelty-Seeking” [*F*(1,45) = 4.175; *p* < 0.05] was significant. Post hoc comparisons showed that only the group of HNS mice conditioned with the high dose of WIN 55212-2 spent more time in the drug-paired compartment in Post-C than during Pre-C (*p* < 0.05). This CPP disappeared after two extinction sessions and was not reinstated by priming with 0.0375 mg/kg of WIN 55212-2. Thus, the HNS trait would seem to increase the sensitivity of mice to the rewarding effects of WIN 55212-2 (see Figure 3). Linear and logistic regression analysis did not show any significant correlation between the novelty-seeking trait and development of the CPP induced by 0.075 mg/kg of WIN 55212-2.

#### 3.1.2. Experiment 2

Experiment 2 is about the effects of exposure of HNS and LNS mice to agonist and antagonist cannabinoids on acquisition of the CPP induced by a subthreshold dose of WIN 55212-2.

**Figure 4:**
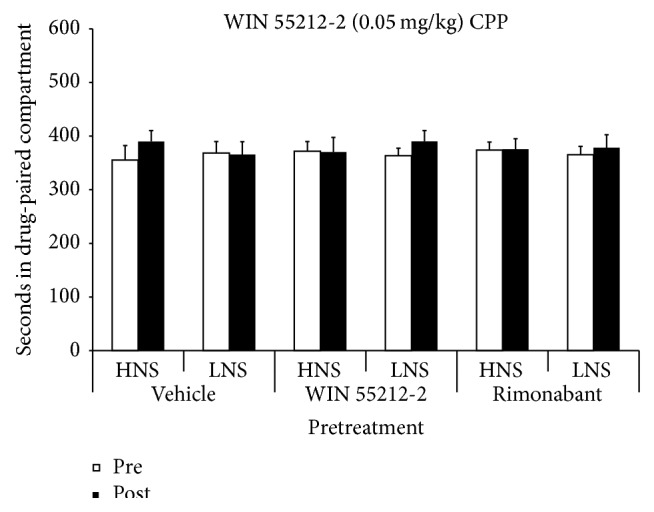
Effects of WIN 55212-2 (0.05 mg/kg) on the CPP paradigm in HNS and LNS adolescent mice pretreated 6 days before initiation of conditioning with vehicle (HNS, *n* = 12; LNS, *n* = 13), 0.1 mg/kg of WIN 55212-2 (HNS, *n* = 12; LNS, *n* = 13), or 1 mg/kg of rimonabant (HNS, *n* = 14; LNS, *n* = 15). Bars represent time in seconds spent in the drug-paired compartment during preconditioning (white) and postconditioning (black).

The ANOVA did not show any significant effect, thus indicating that pretreatment with a cannabinoid agonist or antagonist did not increase the sensitivity of mice to the conditioned rewarding effects of WIN 55212-2 (see Figure 4).

### 3.2. Study 2

Study 2 explains the influence of the novelty-seeking phenotype on the effects of agonist and antagonist cannabinoid on acquisition of the CPP induced by cocaine.

**Figure 5:**
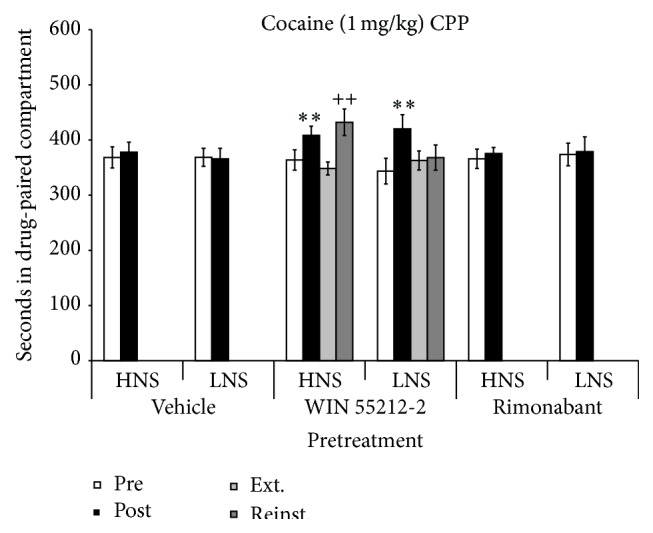
CPP induced by cocaine (1 mg/kg) in HNS and LNS adolescent mice pretreated 6 days before initiation of conditioning with vehicle (HNS, *n* = 15; LNS, *n* = 14), 0.1 mg/kg of WIN 55212-2 (HNS, *n* = 13; LNS, *n* = 12), or 1 mg/kg of rimonabant (HNS, *n* = 14; LNS, *n* = 13). Bars represent time in seconds spent in the drug-paired compartment during preconditioning (white), postconditioning (black), the last extinction session (light grey), and reinstatement (dark grey). Values are mean ± SEM. ^*∗∗*^ *p* < 0.01, difference with respect to the preconditioning session. ^++^ *p* < 0.01, difference with respect to the previous extinction session.

The ANOVA of the data obtained with the mice conditioned with 1 mg/kg of cocaine revealed a significant effect of the interaction “Days × Pretreatment” [*F*(2,78) = 3,952; *p* < 0.01]. Post hoc comparisons showed a significant increase in the time spent by HNS and LNS mice pretreated with WIN 55212-2 in the drug-paired compartment in Post-C with respect to Pre-C (*p*s < 0.01). After extinction of CPP (7 sessions), a priming dose of 0.5 mg/kg of cocaine induced reinstatement of CPP only in HNS mice (*p* < 0.01). Thus, pretreatment with WIN 55212-2 increased the rewarding effects of cocaine irrespective of the novelty-seeking profile of the mice, but only HNS animals were more sensitive to reinstatement after cocaine priming (see Figure 5).

**Figure 6:**
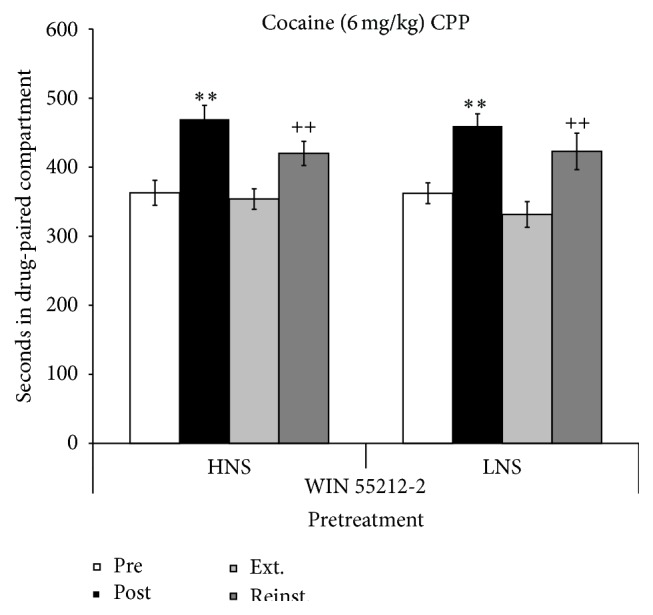
CPP induced by cocaine (6 mg/kg) in HNS and LNS adolescent mice pretreated 6 days before initiation of conditioning with 0.1 mg/kg of WIN 55212-2 (HNS, *n* = 14; LNS, *n* = 15). Bars represent time in seconds spent in the drug-paired compartment during preconditioning (white), postconditioning (black), the last extinction session (light grey), and reinstatement (dark grey). Values are mean ± SEM. ^*∗∗*^ *p* < 0.01, difference with respect to the preconditioning session. ^++^ *p* < 0.01, difference with respect to the previous extinction session.

**Figure 7:**
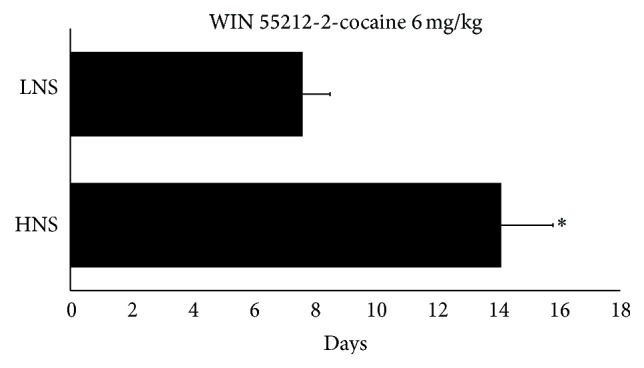
Effects of the novelty-seeking phenotype on the extinction of cocaine CPP. Mean number of days needed to achieve complete extinction of CPP in HNS and LNS mice. After conditioning with 6 mg/kg of MDMA, all groups showed CPP in the Post-C test and underwent daily extinction sessions. HNS mice required more extinction sessions to achieve complete extinction of CPP than LNS mice. ^*∗*^ *p* < 0.05, significant difference with respect to the LNS group.

The ANOVA of the data obtained with the mice pretreated with WIN 55212-2 and conditioned with 6 mg/kg of cocaine revealed a significant effect of the variable “Days” [*F*(3,72) = 17.772; *p* < 0.01], with mice spending more time in the drug-paired compartment in Post-C than during Pre-C (*p* < 0.01) and in the Reinstatement test than during the previous Extinction test (*p* < 0.05). Post hoc comparisons showed a significant increase in the time spent by HNS and LNS mice in the drug-paired compartment in Post-C with respect to Pre-C (*p*s < 0.01) and revealed a reinstatement of CPP (*p*s < 0.05) after a priming dose of 3 mg/kg of cocaine (see Figure 6). The Kaplan-Meier test showed that the time required for extinction was longer in HNS than in LNS mice (14 versus 7 days, *X* ^2^ = 3.995, *p* < 0.05) (see Figure 7).

Linear and logistic regression analysis did not show any significant correlation between the novelty-seeking trait and development of the CPP induced by 1 or 6 mg/kg of cocaine, in accordance with a previous study carried out in our laboratory [42].

## 4. Discussion

Animal models are a vital tool for increasing our understanding of the behavioural traits (e.g., novelty-seeking) and environmental events (e.g., early drug exposure) associated with the individual vulnerability of subjects to repeated drug consumption and how these factors interact to facilitate the development of drug addiction. The results of the present study demonstrate for the first time that adolescent HNS mice are more vulnerable to the rewarding effects of cannabinoids. Even more importantly, given the high risk of adverse effects associated with cocaine, there was some indication that adolescent mice with this phenotype are more vulnerable to the reinstating effects of a low dose of cocaine if they have been previously exposed to a cannabinoid agonist.

The first contribution of this study is the demonstration that the HNS phenotype increases the sensitivity of mice to the conditioned rewarding effects of the cannabinoid agonist WIN 55212-2. We have observed that HNS mice acquire CPP after conditioning with 0.075 mg/kg, a dose that is ineffective in LNS. Although no previous studies have evaluated the influence of the novelty-seeking trait on the rewarding effects of cannabinoids, our results are in accordance with those observed with other drugs of abuse, such as cocaine or MDMA, which have demonstrated that HNS mice are more sensitive to the conditioned rewarding effects of these drugs [41–43]. It is not clear if the ability of HNS mice to develop CPP after administration of the cannabinoid agonist is related with an increase in the reinforcing/rewarding value of this drug for these animals or whether they acquire incentive learning in a more efficient way than LNS mice. Either way, increased levels of incentive salience attributed to drugs and/or drug-associated cues can enhance the intensity and duration of incentive motivation for drugs of abuse (higher unconscious wanting and conscious craving), thus facilitating the transition to drug addiction, as suggested by Robinson and Berridge [49]. It has been reported that HNS animals have a characteristic striatal DA profile (higher endogenous levels, stronger responses to reward cues, and lower availability of D2/D3/D4 receptors [50]), which may contribute to the tendency of these animals to exhibit approach reactions towards novel stimuli and may explain the increased CPP observed in the present study.

The second important result of the present study is that even though exposure to the cannabinoid agonist WIN 55212-2 during adolescence did not enhance the acquisition of CPP induced by WIN 55212-2 itself at the doses tested, it did enhance the acquisition of CPP induced by a low dose of cocaine. Mice pretreated with WIN 55212-2 exhibited CPP after conditioning with a low dose of cocaine that was ineffective in inducing CPP in animals pretreated with vehicle. Moreover, mice pretreated with WIN 55212-2 showed priming-induced reinstatement of the CPP induced by 6 mg/kg of cocaine, an effect that has not been observed in naïve mice [46]. Although clinical and epidemiologic studies show that cannabis consumption usually precedes the initiation of cocaine use [27–29], only two studies have evaluated the effect of stimulation of the endocannabinoid system (ECS) during adolescence on the subsequent effects of cocaine. In line with the results of the present study, adolescent rats pretreated with cannabinoid agonists showed increased locomotor responses to cocaine challenge [38] and a higher rate of cocaine self-administration [35]. THC preexposure also increases the rewarding effects of nicotine [36], morphine [39], and MDMA [40, 47, 51]. Conversely, other studies with adult animals have shown that cannabinoid agonists reduce cocaine reward [52, 53]. Usually, genetic ablation or antagonism of CB1 receptors decreases the self-administration [54–56], CPP [57–60], and sensitization [55, 61] induced by cocaine, although some studies have found no effects [55, 62, 63]. Thus, the ECS plays a complex role in the behavioural effects of different drugs of abuse [64]. The present study extends these results by demonstrating that exposure to a CB1 agonist during adolescence increases the conditioned rewarding effects of cocaine, thus suggesting sensitization of the brain reward system.

ECS plays an important role in adolescent brain development, and the strong stimulation of this system by cannabinoids might induce long-lasting neurobiological consequences, such as alterations in emotional and cognitive performance, increased risk of developing schizophrenia, and enhanced vulnerability to the use of drugs of abuse [22]. In particular, changes in the DA reward system induced by exposure to cannabinoids could underlie the behavioural effects observed. Cannabinoid adolescent exposure induces an upregulation of DAT in the caudate-putamen [31], an increase in D1Rs content in the NAcc shell, and a reduction in the expression of D2Rs in CA1 [31]. These changes can contribute to an increase in the reinforcing/rewarding value of the drug, leading to a greater risk of developing compulsive drug seeking. Moreover, neuroadaptations in the ECS may be part of the neuroplasticity associated with the development of cocaine addiction [65].

On the other hand, neither exposure to the cannabinoid agonist WIN 55212-2 during adolescence nor pretreatment with rimonabant modified the subsequent effect of WIN 55212-2 in the CPP paradigm, in contrast with that observed with cocaine in this study or in previous studies with morphine or MDMA [39, 40]. There is not a clear reason for the absence of an increase in vulnerability to the CPP induced by WIN 55212-2 after adolescent preexposure to this drug. It is possible that the particular profile of WIN 55212-2 in the CPP paradigm underlies the results observed. This drug only induces CPP with specific doses, with higher or lower doses being ineffective [39]. Given that our main objective was to detect differences in the influence of adolescent cannabinoid exposure between HNS and LNS, we used a very low dose of WIN 55212-2 (0.05 mg/kg), as we expected that cannabinoid pretreatment would cause a shift to the right of the dose-response curve. For example, in a previous study we observed that older adolescent mice (PND 52) developed CPP after conditioning with 0.05 mg/kg of WIN 55212-2 [47], suggesting that adolescent maturation is a factor that increases sensitivity to this drug. In any case, it is possible that if higher doses were used during conditioning, we would observe a potentiation of the rewarding effects of WIN 55212-2 in mice pretreated with this cannabinoid during adolescence. In fact, the effects of novelty-seeking phenotype (experiment 1) were only apparent when the dose of WIN 55212-2 was higher (0.075 mg/kg) than the dose used in this experiment. This warrants a tentative conclusion concerning the effects of adolescent exposure to WIN 55212-2 on a subsequent WIN 55212-2 CPP. Future studies using higher doses of WIN 55212-2 during conditioning or different procedures of preexposure to this drug are necessary to determine whether or not adolescent exposure to cannabinoids alters the effects of WIN 55212-2 in the CPP paradigm.

The main result of the present work is that the novelty-seeking phenotype determines the influence of adolescent cannabinoid exposure on the subsequent rewarding effects of cocaine in the CPP paradigm. Not all mice are equally vulnerable to the sensitization of the brain reward system induced by stimulation of the cannabinoid system during adolescence. HNS mice are particularly affected by pretreatment with WIN 55212-2, showing a priming-induced reinstatement of the CPP induced by 1 mg/kg of cocaine and an enhanced duration of the CPP induced by 6 mg/kg of this drug (effects that are not observed in LNS mice exposed to WIN 55212-2). Our results support the idea that exposure to cannabis during adolescence, though it can increase the rewarding effects of subthreshold doses of cocaine six days after pretreatment, is not enough to promote long-lasting brain changes that increase the likelihood of the development of cocaine addiction (which can be evaluated by the maintenance of CPP or its reinstatement after extinction). Genetic and behavioural predispositions—for example, a novelty-seeking phenotype—may underlie increased adolescent drug experimentation, enhanced reward when the subject is exposed to the drug, and the development of neuroadaptations that lead to later adult addiction.

Animal models allow us to answer questions that cannot be explored in human subjects due to ethical constraints and can be useful for analysing possible neurobiological substrates underlying interactions between environmental and biological factors that contribute to individual vulnerability to drug abuse and addiction. The phenotypic causation gateway hypothesis proposes a sequential progression of drug use in which early initiation of cannabis use is a risk factor for the future consumption of other drugs of abuse, such as cocaine [21]. In support of this hypothesis, we have observed that mice exposed to the cannabinoid agonist during adolescence show an increased acquisition and reinstatement of cocaine CPP. The alternative common liability hypothesis proposes that cannabis and use of other illicit drugs are influenced by correlated genetic and environmental factors [66]. Our research has shown that the novelty-seeking trait is associated with an enhanced acquisition of WIN 55212-2 CPP, as we have observed previously with cocaine [42], and also with an increase in the effects of adolescent cannabinoid exposure on reinstatement and maintenance of cocaine CPP. Thus, the results of the present study support the formulation of a “vulnerability” model that integrates the gateway and common liability hypotheses in order to explain the increased likelihood of transition from regular cannabis use to that of other substances (such as cocaine or heroin).

There is a subpopulation of adolescents which engages in extremely risky cannabis and drug use early in life and which seems to run a greater risk of abuse and addiction later in life. The results of the present study suggest that there is not a direct causal mechanism between adolescent drug exposure and the subsequent development of addiction. Instead, there are individual brain and behavioural differences that are present prior to the onset of drug use, such as a higher propensity for sensation-seeking, which influence both the tendency to experiment with drugs of abuse early in life and the later development of addiction. These subjects appear to be more vulnerable to the appearance of permanent neurobiological changes following drug exposure that may lead to the transition from voluntary to compulsive drug use. Thus, specific preventive programs aimed at these more vulnerable subjects could reduce drug consumption and later addiction.
