# Corrigendum to “Effects of Cannabinoid Exposure during Adolescence on the Conditioned Rewarding Effects of WIN 55212-2 and Cocaine in Mice: Influence of the Novelty-Seeking Trait”

**DOI:** 10.1155/2016/6702083

**Published:** 2016-07-28

**Authors:** M. Rodríguez-Arias, C. Roger-Sánchez, I. Villanova, N. Revert, C. Manzanedo, J. Miñarro, M. A. Aguilar

**Affiliations:** Unidad de Investigación Psicobiología de las Drogodependencias, Departamento de Psicobiologia, Facultad de Psicología, Universidad de Valencia, 46010 Valencia, Spain

In the article titled “Effects of Cannabinoid Exposure during Adolescence on the Conditioned Rewarding Effects of WIN 55212-2 and Cocaine in Mice: Influence of the Novelty-Seeking Trait”, [1] the name of the third author was given incorrectly as I. Vilanova. The author's name should have been written I. Villanova. The revised author list is shown above.
